# Somatosensory Functions of Melastatin Transient-Receptor Potential Channels in the Teeth: Molecular Basis for Thermal Dentine Hypersensitivity

**DOI:** 10.3390/dj14050311

**Published:** 2026-05-19

**Authors:** Ramón Méndez, José Martín-Cruces, Marcos Anache, Mirian Teulé-Trull, Yolanda García-Mesa, Patricia Cuendias, José A. Vega, Teresa Cobo

**Affiliations:** 1 Master ENDORE, Universidad de Santiago de Compostela, 15705 Santiago de Compostela, Spain; uo298626@uniovi.es (R.M.); uo298581@uniovi.es (M.A.); mirian-teule.trull@usc.gal (M.T.-T.); 2 Grupo SINPOS, Departamento de Morfología y Biología Celular, Universidad de Oviedo, 33006 Oviedo, Spain; garciamyolanda@uniovi.es (Y.G.-M.); cuendiaspatricia@uniovi.es (P.C.); javega@uniovi.es (J.A.V.); 3 Instituto de Investigación Sanitaria del Principado de Asturias (ISPA), 33011 Oviedo, Spain; 4 Facultad de Ciencias de la Salud, Universidad Autónoma de Chile, Santiago 750012, Chile; 5 Departamento de Cirugía y Especialidades Médico-Quirúrgicas, Universidad de Oviedo, 33006 Oviedo, Spain; 6 Instituto Asturiano de Odontología, 33006 Oviedo, Spain

**Keywords:** melastatin transient-receptor potential channels, trigeminal neurons, odontoblasts, thermal sensitivity, dentine hypersensitivity, pulpitis

## Abstract

Dental pain due to dentine hypersensitivity or pulpitis is characterized by short or lasting episodes of pain triggered by normally innocuous stimuli originating from exposed dentine. Both represent the most frequent pain of the orofacial region. Transient receptor potential (TRP) superfamily of ion channels participates in the detection of different modalities of sensibility in the mammalian sensory teeth system, i.e., trigeminal neurons and odontoblasts. In particular, some members of the melastatin family (TRPM) serve as molecular thermal sensors, and temperature is one of the most potent stimuli in triggering dentine hypersensitivity. Here we review and update the information about the distribution of TRPM channels in the trigeminal ganglion and dental pulp cells, especially odontoblasts, in humans and animal models. In addition to the well-known sensory roles of TRPM, other functions such as the development and mineralization of teeth are considered.

## 1. Introduction

Dentine hypersensitivity is a common odontogenic condition, with a prevalence ranging from 3 to 98% [1], that is the one of the most frequent pains in the orofacial region [2]. It is characterized by short episodes of pain triggered by normally innocuous stimuli, due to exposition of dentine, and therefore dental pulp, which cannot be ascribed to any other teeth damage [3]. The stimuli that can directly or indirectly affect the dental pulp include mechanical, chemical or thermal irritation, dental caries, infiltration of bonding materials, trauma and orthodontic movements [4]. It is especially well known that dental pain may occur when intense thermal stimuli are applied on the surface of a normal intact tooth: the drinking/eating of cold or hot drink/food can induce dental pain [5,6]. As a rule, noxious cold induces transient pain while noxious heat causes lasting pain [7,8].

The stimuli initiating dentine hypersensitivity and toothache are detected by sensory nerves arising from neurons localized in the trigeminal ganglion (TG) that express different molecules that detect more or less selectively specific stimuli [9,10]. The peripheral process of the pseudo-unipolar axon of the trigeminal neurons reaches the orofacial tissues including the dental pulp. Within the dental pulp, trigeminal nerve fibers innervate the odontoblasts through Aδ myelinated and C unmyelinated nerve fibers. The Aδ fibers are principally located at the pulp–dentin border and reach the basal odontoblast layer, while the C fibers enter the dentin tubules [11,12]. Furthermore, a small number of Aβ-fibers myelinated enter the dental pulp too [13]. In addition to nerve fibers, the odontoblasts are now regarded as dentine multi-sensors since they express functional ion channels related to different modalities of sensitivity [14,15,16,17,18]. Gating the odontoblast ion channels induces release of ATP which acts as transmitter between odontoblasts and nerve fibers activating ionotropic ATP receptors, thus initiating the transmission of the stimuli to the central nervous system [19,20,21].

Currently there are three hypotheses proposed to explain dental pain and dentinal hypersensitivity: the first hypothesis is the so-called hydrodynamic theory, and attributes dental sensibility to fluid movement within dentinal tubules. The second hypothesis, known as nervous theory, refers to direct stimulation of dental nerves by different stimuli. The third hypothesis involves the odontoblasts as sensory cells and is supported by functional expression of ion channels of different families by these pulpal cells. According to Solé-Magdalena et al. [3], “*These three hypotheses are not mutually exclusive and cannot be considered separately because of the presence of nerves and odontoblast processes within the dentinal tubules, bathing in the dentinal fluid, and the close apposition of the odontoblasts to the dentinal or basal nerves terminals*”. Thus, chemical composition, mechanical properties (viscosity, osmolarity, velocity or direction) and thermal stimuli (hot and cold) can (a) directly stimulate nerve fibers of the subodontoblastic plexus or the nerves located in dentine tubules, or (b) the odontoblasts which through odontoblast–nerve complexes transmit the stimuli to the nerves. While different mechanisms have been proposed to explain tooth sensitivity (see [3,14] for a review) at present, the key role of ion channels in detection of the different qualities of tooth sensitivity is almost universally accepted.

García-Ávila and Islas [22] define thermosensation as “*the ability of organisms to detect and codify both environmental and internal temperature*”. The detection and transmission of thermal stimuli depend on the activity of various ion channels in the plasma membrane of sensory nerves, including voltage-gated K^+^ channels, voltage-gated Na^+^ channels and depolarizing ion channels that open in response to changes in temperature [23]. This latter type of ion channel is often considered as the primary molecular sensor. Nevertheless, the contribution of temperature-sensitive ion channels to thermosensation is highly dependent on the cellular context, which may explain why some highly thermosensitive ion channels are present in cells that are not involved in thermosensory processes.

In recent decades, members of the transient receptor potential (TRP) superfamily of ion channels have been detected in dental primary afferent neurons, nerve fibers of the dental pulp and odontoblasts where they transduce external stimuli into several signals in the tooth [24]. This review focuses on the melastatin family, which mediate different modalities of sensibility but especially thermosensitivity. Some TRPM channels serve as molecular thermal sensors for cold, warm, and noxious heat [25], and temperature changes are potent stimuli in triggering dentine hypersensitivity [1,4,26].

## 2. Material and Methods

A narrative review of the scientific literature on TRPM ion channels and tooth sensitivity, as well as another on TRPM channel generalities, was carried out in the PubMed and Google Scholar databases. The keywords used were teeth, tooth, trigeminal ganglia, dental pulp, odontoblasts, TRPM ion channel, TRPM* ion channel, and the Boolean operators AND, OR, NOT., with no time limitation of articles written in English. The type of study was also not limited. The selection of the articles was made by reading the title and the abstract and reviewing the full text. Studies in humans, animal models, and cell lines were included. The selected studies were grouped according to general data on TRPM channels, presence of TRPM channels in the trigeminal ganglion and nerve fibers of the dental pulp, presence of TRPM channels in odontoblasts and other cell types of the dental pulp. Moreover, the non-sensory roles of TRPM ion channels in teeth were selected. It should be noted that, given the narrative nature, there may be bias in the selection of studies.

## 3. The Superfamily of TRP Channels

TRP superfamily of ion channels consists of 28 integral transmembrane proteins that function as non-selective cation channels; few are highly Ca^2+^-selective and some are permeable for highly hydrated Mg^2+^. TRP channels are subdivided into seven subfamilies according to amino acid sequence homology: TRPA (ankyrin), TRPC (canonical), TRPM (melastatin), TRPML (mucolipin), TRPP (polycystin) and TRPV (vanilloid). On the other hand, based on their sequence and topological features, TRP genes are divided into Group 1 (TRPC, TRPV, TRPM, TRPA, and TRPN), and Group 2 (TRPP and TRPML). TRP channels show a variety of gating mechanisms with modes of activation ranging from ligand binding, voltage and changes in temperature to covalent modifications of nucleophilic residues [27,28,29,30].

TRP ion channels consist of four subunits resulting in homomeric or heteromeric channels [31]. They share some structural characteristics including a three-dimensional structure with six transmembrane segments (S1 to S6), N- and C-terminal cytoplasmic domains, and a small α-helix loop between S5 and S6 segments that form the channel pore, while S4 corresponds to a voltage-sensor-like domain, capable of sensing changes in intracellular ion concentration. The N- and C-terminal cytoplasmic domains are of variable length and contain residues and regulatory motifs unique for each family. TRP differs from other voltage-gated channels by the aminoacidic sequence of their subunits, which confers to them differential biophysical characteristics and response to different exogenous and endogenous modulators. TRP channels have a ubiquitous expression and are involved in very heterogeneous physiological processes as well as in several pathological conditions [32,33,34,35,36].

## 4. TRP Melastatin (TRPM) Ion Channels: An Overview

**Figure 1:**
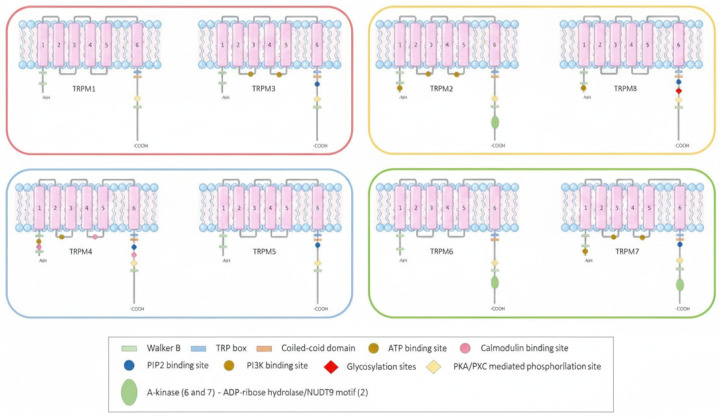
Channel structure of the transient receptor potential melastatin (TRPM) subfamily. The channel domain contains six transmembrane segments (1–6), 1–4 corresponding to a voltage-sensor-like domain; the pore is formed by the loop between the 5 and 6 segments. The N-terminus is composed of four melastatin homology regions and the C-terminus is composed of TRP and the coiled-coil. Based on the homology sequence of the coiled-coil in the C-terminus, the TRPM subfamily is divided into four groups: TRPM1/TRPM3 (red box), TRPM2/TRPM8 (yellow box), TRPM4/TRPM5 (blue box) and TRPM6/TRPM7 (green box). Inspired by Huang et al. (2020) [41].

The TRPM channel subfamily consists of eight members (TRPM1-8) of non-selective cation channels that share common structural characteristics with other TRP channels but characteristically have a large cytoplasmic, an N-terminal melastatin homology region (MHR) domain, six transmembrane helices (S1–S6), a TRP helix, and a complex C-terminal domain. They assemble as tetramers, forming a central pore for cation permeation. In addition, some TRPM channels feature unique enzymatically active domains. Based on the homology sequence of the coiled-coil in the C-terminus, TRPM channels are grouped in four pairs: TRPM1 and TRPM3; TRPM2 and TRPM8; TRPM4 and TRPM5; and TRPM6 and TRPM7 [37,38,39,40,41,42,43,44]; [Figure 1].

The mechanisms of activation of TRPM channels vary largely among members [37,44]. However, more than half are sensitive to a wide range of temperatures, from cold to hot, and exhibit distinct thermal activation thresholds: <17 °C for TRPM1, 38 °C for TRPM2, >40 °C for TRPM3, 15 °C to 35 °C for TRPM4 and TRPM5, and <20–28 °C for TRPM8 [45,46]. Furthermore, some TRPM channels also respond to mechanical stimuli [47], redox status, intracellular calcium (with TRPM4 and TRPM5 being the only non-calcium-conducting channels [48,49]), or ligands such as menthol [50,51].

The expression of TRPM channels is highly variable. Some of these include TRPM7, while others are restricted to some tissues and organs. They have been detected in the central and periphery nervous system, including the retina. Outside the nervous system they were detected in the prostate, ovary, kidney, intestine, pancreas, heart and blood vessels, melanocytes, pituitary, bone, and adipose tissue [52,53,54].

The preceding paragraphs intend a brief presentation of TRPM channels. There are excellent recent reviews regarding these ion channels under normal and pathological conditions [22,24,47,55,56], including those contained in this Special Issue of *International Journal of Molecular Science*, and we refer to those articles to the interested in the field. The pages that follow relate directly to the distribution and function of TRPM channels in the tooth sensory system; to them, that is, the neurons of the trigeminal ganglion and their projections to the teeth and the odontoblasts regarded as sensory cells.

## 5. Distribution of TRPM Ion Channels in Teeth and Trigeminal System

### 5.1. Trigeminal Ganglion and Pulpal Nerve Fibers

The primary sensory neurons of the trigeminal ganglion as well as sensory cells in the dental pulp, especially the odontoblasts, express members of the TRPM family of ion channels at the mRNA or protein levels. It must be noted that although each ion channel is associated with the detection of one quality of sensibility, most neurons and odontoblasts express more than one ion channel. Thus, the capacity exhibited to preferentially detect specific stimuli is the result of a characteristic combinatorial expression of different ion channels [3].

The expression of TRPM channels by trigeminal neurons is highly variable. This heterogeneity may be due to species-specific differences, but it may also be related to the sensitivity of the techniques used for its detection.

In mice, among the 28 TRP channel genes identified in mammals, 17 have been detected in the mouse trigeminal ganglion at the mRNA level, including all members of the TRPM family, with the exception of TRPM1 [57]. As far as we know, TRPM1 has never been detected in the somatosensory system of the teeth and is preferably expressed in the retina [58].

Other studies also demonstrate the occurrence of individual TRPM channels in the trigeminal neurons of mice. They include TRPM3 [59], and TRPM8, which is present in 5.7% of neurons, innervating the dental pulp [60] highly co-localized with TRPV1 and Piezo2 [61].

In the rat trigeminal ganglion about 50% of neurons, mostly of small-to-medium-sized cell bodies, express TRM3 but only approximately 20% innervate the tooth pulp while the others are destined for other orofacial regions [62]. TRPM8 is also expressed in small-diameter neurons, the percentage of which being 15%, and is sometimes co-localized with TRPA1 [63,64,65,66,67].

TRPM2, TRPM3, TRPM7 and TRPM8 have also been detected in the neurons of the human trigeminal ganglion using different techniques [68]. 

But the detection of temperatures by neurons in the trigeminal ganglion by activation of heat-sensitive ion channels is by no means simple. Closely related to TRPM8 is TRPA1 (a channel of the TRP superfamily that has not been considered in this review) which also detects cold. These two cold-sensitive ion channels are expressed in trigeminal ganglion neurons innervating the dental pulp but do not participate in dental pulp sensitivity to cold. On the other hand, other research does implicate TRPA1 and TRPM8 in the detection of cold, and it has also been shown that in the trigeminal ganglion there is a subset of neurons that respond to the detection of cold by a mechanism independent of TRPA1 or TRPM8 [69,70]. On the other hand, TRPA1 and TRPM8 were co-expressed with TRPV1 (a hot sensor ion channel) in dental afferent neurons, suggesting an ambiguity between cold and hot stimuli-induced tooth pain [65]. A recent study suggested that temperature sensation has redundant mechanisms for detection, although whether dental sensory systems utilize a similar mechanism has not been demonstrated [71]. It is clear that more studies are needed to clarify the role of these ion channels in dental temperature sensation and sensitivity.

**Figure 2:**
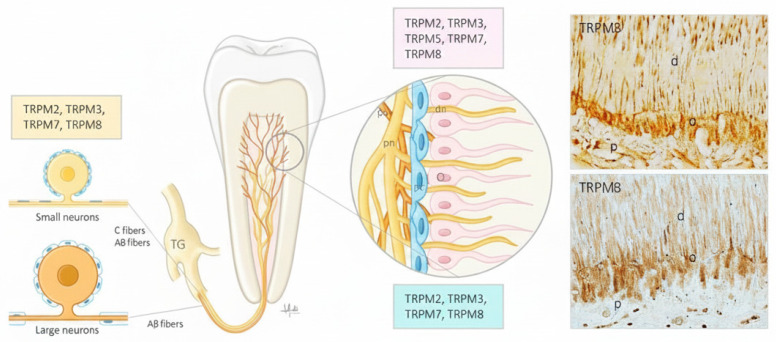
Schematic representation of tooth innervation and expression of TRPM ion channels in the trigeminal ganglion (TG) neurons, odontoblasts and fibroblasts of the tooth pulp. The channels indicated in red have been detected in humans. O: odontoblasts, dn: dentinal nerves, pn: pulpal nerves. The photographs on the right correspond to sections of human teeth showing the expression of TRPM3 and TRPM8 in the odontoblasts; M3 was also detected in nerve profiles of the dental pulp. d: dentin, o: odontoblast, p: dental pulp.

The results are summarized in Table 1 and Figure 2.

### 5.2. Odontoblasts and Pulpal Cells

The detection of TRPM channels in odontoblast and other pulpal cells has been largely studied in the human teeth, different animal models and isolated cells in vitro.

TRPM3 is expressed in primary cultured mouse odontoblasts [72] and TRPM5 was found in mouse odontoblasts asymmetrically distributed, localizing proximal to and within odontoblast processes [73]. Also, TRPM7 has been detected in most odontoblasts of adult rats, predominantly in the odontoblastic process region [72,73,74,75,76]. Results on TRPM8 are more contradictory. In fact, while a study did not detect it in both acutely isolated rat odontoblasts and in pulpal slice-derived odontoblasts [72,77], another showed TRPM8 in acutely isolated adult rat odontoblasts cultured from pulpal slices [78]. The reason for these discrepancies is probably related to the growing conditions.

Regarding humans, TRPM2 was detected in dental pulp fibroblasts and odontoblasts [79]. In cultured odontoblast-like cells, in native human odontoblasts and dental pulp fibroblasts, TRPM8 has been detected [80,81,82]. Nevertheless, detection of TRPM8 failed in human immortalized dental pulp cells derived toward an odontoblast phenotype in vitro [83,84]. Again, the differences in the results could be related to the sensitivity of the study methods used.

Most of those channels, but not all [72,73,74,75,76,77], were found to be functional in pulpal fibroblasts and odontoblasts since increased intracellular calcium ([Ca(^2+^)](i)) was observed in response to the agonist or temperature stimuli, and the responses were blocked with specific antagonists [72,78].

The results are summarized in Table 2 and Figure 2.

### 5.3. Other Sensibilities

The odontoblasts presumably mediate the early stage of sensory processes in teeth, detecting mechanical, thermal, and chemical sensing [7,12,18]. Although this review was centered in the thermosensor role of TRPM channels, several members of this family also exhibit mechanosensitivity. TRPM3 [72] and TRPM7 [76] mediate osmo- and mechano- sensitivity in odontoblasts. TRPM8 is also involved in hyperosmolar sweet foods’ dentin hypersensitivity [62].

Interestingly, an intriguing relationship exists between thermal and mechanical stimuli in teeth. Thermal stimulation on the tooth surface can induce fluid movement in the dentinal tubules because of thermal expansion or contraction of the fluid, and tooth structures respond to this thermal gradient with mechanical stress and deformation of the dentine [85]. These data suggest that temperature may exert mechanical stress on the odontoblasts as well as on the pulpal tissues. In turn, the mechanical stresses may directly activate the mechanosensitive TRP channels along with other mechanosensors present in the odontoblasts and pulpal nerve fibers within/near the dentinal tubules.

## 6. Non-Sensory Functions of TRPM Channels in Teeth

In addition to the function of TRPM channels in tooth sensibility, they also perform non-nervous roles within the teeth, especially during development. TRPM channels are expressed in odontoblasts and other dental pulp cells (including stem cells), and ameloblasts, which support a wide range of functions.

Odontoblasts organize and regulate the synthesis of the mineralized dentin matrix [86,87,88]. So, TRPM7 in odontoblasts plays an important role in dentine formation and mineralization by regulating intracellular Mg^2+^ and alkaline phosphatase activity [75,76,89].

On the other hand, TRPM7 is highly expressed in ameloblasts during tooth development and potentially contributes to the enamel matrix mineralization [90,91]. Consistently, TRPM7-deficient mice show low-volume, opaque, white-colored enamel [92]. Furthermore, TRPM7 also participates in the regulation of human dental pulp stem cells’ proliferation, migration and osteogenic differentiation and may play a role in the dental pulp repair process [93].

Finally, TRPM4 is expressed in rat dental follicle stem cells where it presumably participates in the inhibition of osteogenesis [94].

## 7. TRPM Channels and Dental Pathologies

There is still scarce evidence for the involvement of TRPM ion channels in dental pathologies, but some evidence suggests that it participates in dentin sensitivity, pulp pain and pulpitis to heat and cold and in pain of dentinal origin. This is the case for TRPM2, TRPM3 and TRPM8.

Under pathological conditions such as inflammation, sunburn, or tissue injury, temperatures in the heat range lower than 43 °C stimulate TRPM2 and TRPM3 channels so that they are perceived as painful [28]. TRPM2 is activated by heat cellular stress and regulates cytokine production, cell motility and cell death [95,96] and is implicated in pathogenic pain [97]. Consistently, its expression is increased in the pulpal fibroblasts of teeth with signs of irreversible pulpitis [79].

TRPM3 is involved in inflammatory hyperalgesia and heat-associated inflammation [98,99], although this function is carried out in combination with other ion channels, such as TRPV1 and TRPA1 [71,100,101]. However, it is likely that other ion channels are also involved in thermosensitivity [102]. TRPM3 is also involved in mediating the orofacial analgesic effects of nifedipine, although it has not been shown to be involved in dental pain [103].

It is well known that pulpitis pain might be triggered by the direct activation of cold-responsive thermoreceptors [98], although there are doubts about this [104]. TRPM8 is involved in the painful hypersensitivity that is a symptom of inflammation and neuropathy [105]. A large number of studies have involved TRPM8 in thermal and mechanical hyperalgesia [106,107,108,109,110,111,112,113,114]. Confirming this role, TRPM8 agonists have analgesic properties in acute and chronic pain models [110,111]. However, in animal models, peripheral and central activation of TRPM8 has also been shown to be followed by analgesia [115]. Further studies are needed to clarify the role of TRPM8 in inflammatory and neuropathic pain. TRPM8 is probably also involved in the activation of TRPM8 of pulp nerve fibers by bacterial lipopolysaccharide [116,117] in the course of infectious pulpitis, which can be an extremely painful condition and is often associated with intense lingering pain to thermal stimuli.

## 8. Concluding Remarks

The molecular mechanisms of pulp sensitivity involve the interaction of trigeminal nerve afferents, odontoblasts and to a lesser extent other pulp and immunocompetent cells. Among the mediators of all qualities of dental somatosensitivity are the ion channels of the TRP superfamily [18,118,119,120,121,122,123], and those of the TRPM family are particularly involved in temperature detection [37,40,43]. TRPM channels are widely distributed in the somatosensory system of the teeth, both in the neurons of the trigeminal ganglion and in the odontoblasts [3,14].

Most of the data on the physiology and pathophysiology of TRPM ion channels in teeth comes from studies in animal models, but they surely cannot be exported directly to humans. However, these data suggest that TRPM channels are links between temperatures, inflammation and nociception. Despite this, these studies have opened up potential therapeutic opportunities to treat temperature-related dental pathologies (see [122,123,124]). Pharmacological compounds that interact with TRPM channels both in animal models and humans [35,124] have provided evidence that modulation of those channels will provide new therapeutic approach in the treatment of some pathologies [47]. However, more research is still needed to assess the therapeutic potential of these channels as targets in the pathologies of dental pulp.

## Figures and Tables

**Table 1:** Trigeminal ganglion: dental afferent neurons.

Channel	Percent	Species	Methods	Reference
**TRPM2**		Mouse	PCR	Vandewauw et al. (2013) [57]
		Human	PCR, ma, IIHC	Flegel et al. (2015) [68]
**TRPM3**	Subset sØ	Mouse	PCR, *Ih*	Vriens et al. (2011) [59]
	20%	Rat	IHC	Yajima et al. (2019) [62]
		Mouse	PCR	Vandewauw et al. (2013) [57]
		Human	PCR, ma, IIHC	Flegel et al. (2015) [68]
**TRPM4**		Mouse	PCR	Vandewauw et al. (2013) [57]
**TRPM5**		Mouse	PCR	Vandewauw et al. (2013) [57]
**TRPM6**		Mouse	PCR	Vandewauw et al. (2013) [57]
**TRPM7**		Mouse	PCR	Vandewauw et al. (2013) [57]
		Human	PCR, ma, IIHC	Flegel et al. (2015) [68]
**TRPM8**		Rat	Ca^2+^m	Thut et al. (2003) [64]
	Small neurons	Rat	IHC	Abe et al. (2005) [63]
		Rat	*ih*, IHC	Kobayashi et al. (2005) [65]
	13%	Rat	PCR, IHC	Park et al. (2006) [66]
	58%	Rat	RL, IHC	Kim et al. (2011) [67]
		Mouse	PCR	Vandewauw et al. (2013) [57]
		Human	PCR, ma, IIHC	Flegel et al. (2015) [68]
	5.7%	Mouse	RL, IF	Michot et al. (2018) [60]
	Subset	Mouse	PCR	Lee et al. (2020) [61]

Ca^2+^m: Ca^2+^ microfluorimetry; IF: immunofluorescence; *ih*: in situ hybridization; IHC: immunohistochemistry; ma: microarray; RL: retro labeling, sØ: small diameter.

**Table 2:** Odontoblasts and other dental pulp cells.

Channel	Cell	Species	Methods	Reference
**TRPM2**	Od, pf	Human	IHC	Rowland et al. (2007) [79]
**TRPM3**	Od	Mouse	PCR, Ca^2+^m, EF	Son et al. (2009) [72]
	Od	Rat	PA	Won et al. (2018) [76]
**TRPM5**	Od	Mouse	FC-CGT	Khatibi Shahidi et al. (2015) [73]
**TRPM6**	Od	Rat	PCR	Won et al. (2018) [76]
**TRPM7**	Od (87%)	Rat	PCR, IHC	Kwon et al. (2014) [74]
	Od	Rat	PCR	Won et al. (2018) [76]
**TRPM8**	ObC	Mouse	PCR, Ca^2+^m, EF	Son et al. (2009) [72]
	Od	Rat	PCR	Yeon et al. (2009) [77]
	Od, pf	Human	IHC, WB, EM	El Karim et al. (2011a,b) [80,81]
	Od	Rat	PA, IHC	Tsumura et al. (2013) [78]
	HDPCs	Human	PCR, IHC	Tokuda et al. (2015) [82]
	Od	Rat	PCR, IHC	Tokuda et al. (2015) [82]
	MOLCs	Mouse	PCR, IHC	Tokuda et al. (2015) [82]
	Od	Human	PCR	Tazawa et al. (2017) [83]

HDPCs: human dental pulp cells; MOLCs: mouse odontoblast lineage cells; Od: odontoblast; pf: pulpal fibroblasts—Ca^2+^m: Ca^2+^ microfluorimetry; EF: electrophysiology; EM: electron microscopy; FC-CGT: fluorescent color-coding genetic tracing; IHC: immunohistochemistry; PA: pharmacological approach; WB: Wester botting.

## Data Availability

No new data were created or analyzed in this study. Data sharing is not applicable to this article.
